# Artificial intelligence in the diagnosis of COVID-19: challenges and perspectives

**DOI:** 10.7150/ijbs.58855

**Published:** 2021-04-10

**Authors:** Shigao Huang, Jie Yang, Simon Fong, Qi Zhao

**Affiliations:** 1 Cancer Centre, Institute of Translational Medicine, Faculty of Health Sciences, University of Macau 999078, Macau SAR, China.; 2 Department of Computer and Information Science, University of Macau 999078, Macau SAR, China.; 3 Chongqing Industry & Trade Polytechnic 408000, Chongqing, China.

**Keywords:** Artificial intelligence, COVID-19, diagnosis, deep learning, machine learning

## Abstract

Artificial intelligence (AI) is being used to aid in various aspects of the COVID-19 crisis, including epidemiology, molecular research and drug development, medical diagnosis and treatment, and socioeconomics. The association of AI and COVID-19 can accelerate to rapidly diagnose positive patients. To learn the dynamics of a pandemic with relevance to AI, we search the literature using the different academic databases (PubMed, PubMed Central, Scopus, Google Scholar) and preprint servers (bioRxiv, medRxiv, arXiv). In the present review, we address the clinical applications of machine learning and deep learning, including clinical characteristics, electronic medical records, medical images (CT, X-ray, ultrasound images, etc.) in the COVID-19 diagnosis. The current challenges and future perspectives provided in this review can be used to direct an ideal deployment of AI technology in a pandemic.

## Introduction

Coronavirus disease 2019 (COVID-19) was firstly reported in December 2019 1-3. It has caused a large number of deaths and negatively impacted people's lives worldwide, with more than 100 million confirmed cases of the new coronavirus (SARS-Cov-2) and more than 200 million cumulative deaths worldwide as of late January 2021^4^, ^5^. The patients experience flu-like symptoms such as fever, dry cough, tiredness, difficulty breathing. In more severe cases, the SARS-CoV-2 infection often causes fatal pneumonia in the patients 6. Although the rapid progress in vaccines, the epidemic continues to spread out more than 200 countries and regions. In some countries, people have to face new lifestyles to combat COVID-19 7. Therefore, there is still an imminent need to develop antiviral drugs and medical practices to cure COVID-19 patients 5. Many researchers from all over the world are seeking the effective techniques to cope with such challenges 8, 9.

In severe infectious disease outbreaks, both hospitals and physicians are suffered with the increasing workloads that weaken their ability to classify and hospitalize the suspected patients. The previous reports indicated that some of patients with early coronaviral infection were negative for CT 10, limiting the ability of radiologists to reliably rule out disease. While waiting 4-48 hours for confirmation of SARS-CoV-2 coronavirus by RT-PCR, the infected patients may transmit the virus to the close contacts if insufficient resources are used to separate positive patients with other suspected cases. In one report, the hospital-acquired infection was inferred in approximately 40% of cases 11. It is imperative to speedily confirm patients for COVID-19 because initial false negative cases may increase the risk of transmission of the virus to others.

**Figure 1:**
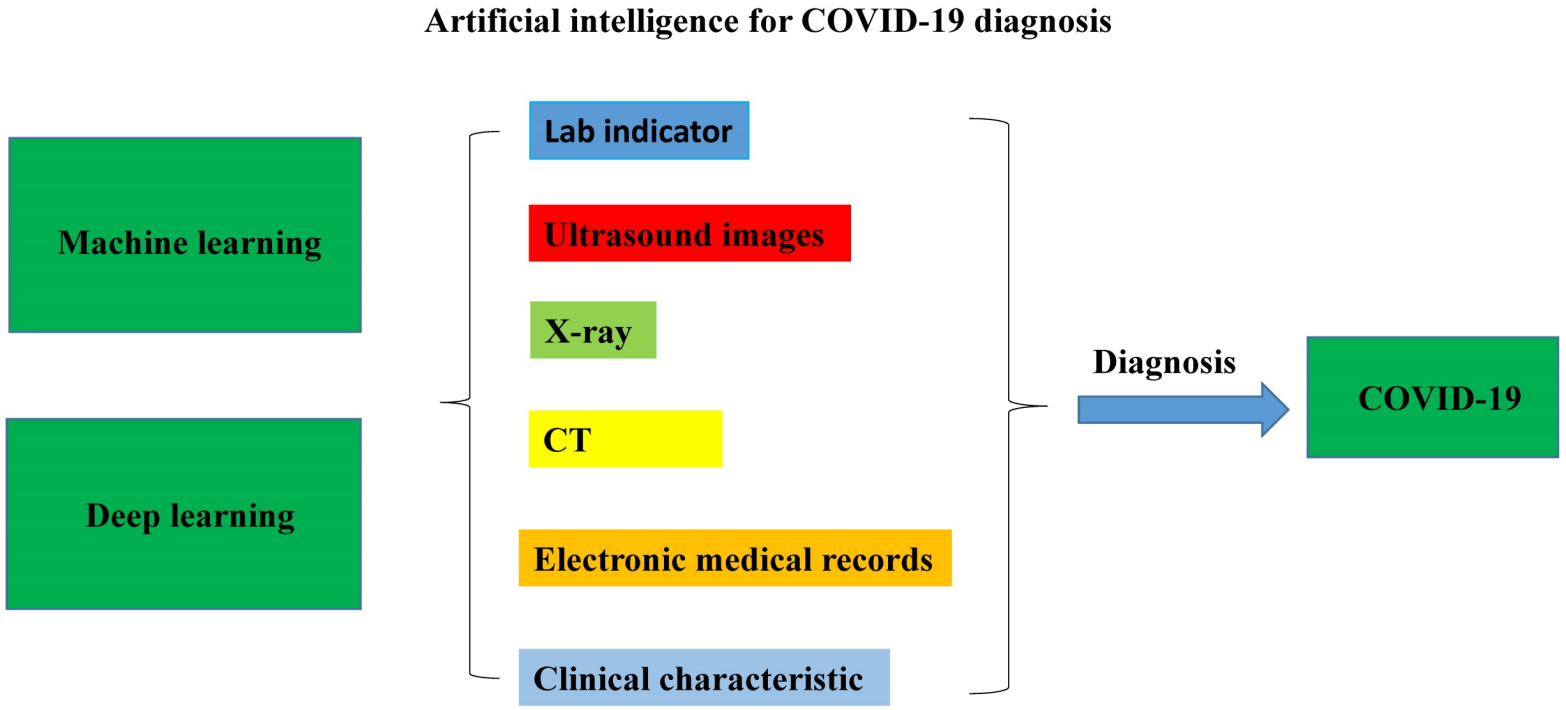
A flowchart of the artificial intelligence methods for COVID-19 diagnosis: machine learning and deep learning were applied in the medical characteristic to diagnosis the COVID-19 infection.

Artificial intelligence (AI) has been deployed at various levels of the health care system, including diagnosis, public health, clinical decision making, and therapeutics 12, 13. Particularly, AI algorithm plays an important role in the fast detection of COVID-19 patients during the current pandemic 14. The number of studies using AI techniques to diagnose COVID-19 rapidly increased in 2020. Most reviews focus on describing diagnosis of COVID-19 from chest CT images using AI technology 15. Therefore, we will comprehensively review the applications of AI for rapid diagnosis of COVID-19 with different medical analyses as well as discuss their challenges and perspectives in COVID-19. As shown in **Fig. 1**, it is mainly compiled from two aspects: machine learning (ML) and deep learning (DL), including electronic medical records, and medical images (CT, X-ray, ultrasound images, etc.), in clinical COVID-19 diagnosis.

## Machine learning-based diagnostic applications

The potential applications of ML for COVID-19 have been previously described 14, 16-26. The details are summarized in **Table 1**.

The first priority of ML was suggested as technical support for early detection and diagnosis of infections. A recent study demonstrated that the more accurate diagnosis could be generated using a computational model trained on large clinical datasets 16. An association between males and higher serum lymphocyte and neutrophil levels was identified by applying ML to reanalyze COVID-19 data from 151 published studies. The COVID-19 patients could be classified into three clinically relevant subtypes based on serum levels of immune cells, gender, and reported symptoms. A sensitivity of 92.5% and a specificity of 97.9% were achieved to discriminate COVID-19 patients from influenza patients using a computational classification model. another study reported that early identification could be performed by a ML model based on the clinical symptoms without CT images at the time of fever clinic admission 17. Peng M et al reported that 18 diagnostic indicators for COVID-19 were highly associated with a significant diagnosis of COVID-19 using AI screening, which improved the accuracy of the clinical diagnosis 18. Chen et al described a ML random forest model used to classify COVID-19 clinical types, which achieved >90% predictive accuracy 19. Zoabi et al generated a ML model that trained on data from 51831 tested individuals in Israel. This model resulted in high accuracy using only eight binary features 20. A combination of seven ML algorithm based on data from UCLA Health System in United States was established to diagnose COVID-19 in the inpatient setting 21. In the test set (n=392), the combined model achieved excellent diagnostic metrics compared to RT-PCR. An et al developed five ML algorithms for death prediction in a case dataset that was provided by the Korean National Health Insurance Service (KNHIS) 5. In prediction of mortality, the sensitivity and specificity exceeded 90% while the areas under the curves (AUC) exceeded 96%.

Chest CT has been used to evaluate the patients with suspected SARS-CoV-2 infection. AI system had equal sensitivity as compared to a senior thoracic radiologist. The radiologists with less expertise in chest imaging demand AI-assisted screening. In a test set of 279 patients, an AI algorithm combining chest CT presentation correctly identified 68%, while radiologists classified all of these patients as COVID-19 negative 14.

Developed ML models are suitable for surveillance efforts to predict the SARS-CoV-2 infection risk of patients with severe diseases. Monahan et al reported that two unique ML models were generated to predict the risk of the hemodialysis (HD) patients having the undetected SARS-CoV-2 infections 27. Through analyzing more than ten thousand patients, they identified top predictor of an HD patient having a SARS-CoV-2 infection in the prior week.

## Deep learning-based diagnostic applications

DL, as a subset of ML, has been explored extensively in the diagnosis of COVID-19, especially in the field of lung detection images, including CT images, X-ray images and ultrasound images. The details are summarized in **Table 2**.

Wu et al developed a DL-based screening framework for coronavirus through a multi-view chest CT imaging 28. The framework trained on multi-view images of chest CT images from two different hospitals in China through a Convolutional Neural Network (CNN) variant, ResNet 50, with a total dataset of 495 patient images, including 368 confirmed cases and 127 images of suspected pneumonia cases. Based on DL method, the proposed diagnosis model showed 76% accuracy, 81.1% sensitivity, 61.5% specificity, and 81.9% AUC. Ardakani et al developed a variant system based on CT images in combination with the CNN architectures for COVID-19 diagnostic detection in which ten convolutional neural networks were used to discriminate positive COVID-19 infections from non-infection groups. Among all networks, ResNet-101 showed the best performance with 99.51% accuracy, 100% sensitivity, 99.4% AUC, and 99.02% specificity 29. Cifci et al diagnosed infections from CT images used AlexNet and Inception-V4, pre-trained models that are widely used in medical image analysis 30. The dataset consists of a public database of 5800 CT images (4640 CT images, with 4640 training samples and 1160 test sets). The dataset analysis has shown a sensitivity of 94.74% and a specificity of 87.37%.

Hybrid systems show the higher accuracy than a single model. Hasan et al generated a hybrid system that combined Q-deformed entropy and DL features (QDE-DF) with LSTM from extracting deep features of CT images 31. The dataset contains 321 chest CT samples, including 118 of COVID-19 cases images, 96 images of pneumonia cases, and 107 images of healthy individuals. As the result, 16 core attributes were extracted by the proposed composite model. The analysis achieved 99.68% accuracy in the training and test sets in the ratio of 7:3.

Multitask DL based model can be used to detect COVID-19 lesions on CT scans. A multitask DL model, including segmentation, classification and reconstruction, was used to detect COVID-19 patient and segment COVID-19 lesion from chest CT images 32. The proposed model was used to analyze a dataset of 1369 patients including 449 patients with COVID-19, 425 healthy individuals, 98 with lung cancer and 397 cases of other diseases. The model had an accuracy of 86%, a sensitivity of 94%, a specificity of 79%, and an AUC of 93%.

Moreover, transfer learning has been applied for the early diagnosis of coronaviruses based on X-ray imaging. Apostolopoulos and Bessiana proposed a system for the automatic diagnosis of COVID-19 cases in which five CNN variants (VGG19, MobileNetv2, Inception, Xception, and Inception-ResNetv2) were used to analyze a dataset of X-ray images from patients with common bacterial pneumonia, confirmed Covid-19 disease, and normal incidents 33. The dataset analysis suggests that DL with X-ray imaging has a high accuracy, sensitivity, and specificity (96.78%, 98.66%, and 96.46%, respectively). A generative adversarial network (GAN) with deep transfer learning has been proposed for coronavirus detection in chest X-ray images 34. The total number of X-ray images in the collection was 307 and contained four categories: COVID-19, normal, pneumonia bacteria, and pneumonia virus. The models contain the Alexnet, Googlenet, and Restnet18. The accuracy of selecting Alexnet as the primary deep transfer model can reach 80.6% when four categories are included, while the accuracy of selecting Googlenet as the primary model can reach 85.2% when three classes are included. Another COVID-19 diagnosis-Net based on an X-ray image was proposed by Ucar and Korkmaz 35. Image data from three public datasets obtained 98.26% accuracy, 98.25% specificity, and 97.39% F1-score in the proposed system. In another study, a DarkNet model for automatic COVID-19 detection using chest X-ray images has been developed 36. The DarkNet model is a classifier used as a "you only look-once" (YOLO) real-time object detection system. The proposed model was evaluated for binary classification (COVID vs. No-Findings, the classification accuracy of 98.08%) and multi-class classification (COVID vs. No-Findings vs. Pneumonia. It has shown a classification accuracy of 98.08%. In addition, the generated heatmaps can assist the clinicians to locate the affected regions on chest X-rays.

Lung Ultrasonography (LUS) has been used for the detection and management of acute respiratory disorders. A recent study shows that DL techniques may assist clinician for the analysis of LUS images from COVID-19 patients 37. A model with three different tasks on LUS imaging: frame-based classification, video-level grading and pathological artifact segmentation, was proposed in the diagnosis of COVID-19. It demonstrated accurate prediction and localization of LUS imaging biomarkers in COVID-19 patients.

## Challenges and perspectives

AI has great potential and opportunity for rapid analysis of large amounts of data. It has played an important role in the prevention of COVID-19 outbreak 38. AI models may be as accurate as experienced radiologists to diagnose COVID-19 38.

It is noteworthy that although some patients infected with COVID-19 are asymptomatic, they have the potential to become transmitters of the virus 6, 39. Although the infection can be confirmed by a polymerase chain reaction, COVID-19 patients with pneumonia symptoms may show a pattern on chest X-ray or CT images that are only moderately characteristic for the clinicians 40. It is difficult to find people who are currently infected with COVID-19 but are asymptomatic 41. The transmission rate of COVID-19 is determined with the ability to confidently recognize infected patients with low false-negative rates. Meanwhile, an effective control of false positives can avoid unnecessary quarantine of patients and thus further reduce the burden on the health care system.

Biomedical imaging (chest X-ray, CT scan, and ultrasound images, etc.) enables to visualize symptoms of pneumonia. Image processing techniques are attractive in the areas of biomedicine and cancer diagnosis 42. It is well known that AI-based biomedical image diagnosis has achieved remarkable success. ML and DL methods have become valuable tools for the discovery of various diseases 40, 43-45. For example, although some patients have already infected by SARS-Cov-2, they show the normal chest CT images. Therefore, the negative predictive value of chest CT images is limited and does not completely rule out infection. The accuracy of the solo AI diagnosis is still challenged. Therefore, to meet clinical needs, AI algorithms is required to combine chest imaging with clinical symptoms, exposure history, and laboratory tests in the diagnosis of COVID-19.

False negative rate is usually high with the laboratory tests, such as nucleotide RT-PCT 46. Medical image screening can provide an intuitive and accurate diagnosis when it is used as the assisted testing method for COVID-19 47. In some epidemic countries, such China and United States, the AI model to diagnose negative sensitivity with CT has been used in the early infections 38. This new strategy provides spare capacity for CT and X-ray imaging scans with the advantages of rapid COVID-19 diagnosis.

## Conclusion

AI model may be as accurate as experienced physicians at diagnosing COVID-19. In this review, we discuss the challenges and perspectives of ML and DL in the COVID-19 as well as the need for further research. The clinical application of AI in the diagnosis of COVID-19 is promising, and additional extensive research is required.

## Figures and Tables

**Table 1:** Application of machine learning-based COVID-19 diagnosis

Authors	Countries	Data Sources	No. of Patients	Techniques	Performances
An et al.^5^	Korea	KNHIS	10237	LASSO, LSVM, SVM with radial basis function kernel, RF, KNN	Sensitivities (90.7% [95% confidence interval: 83.3, 97.3] and 92.0% [85.9, 98.1], respectively) Specificities (91.4% [90.3, 92.5] and 91.8%, [90.7, 92.9], respectively) AUC (0.963 [0.946, 0.979] and 0.962 [0.945, 0.979], respectively)
Zoabi et al.^20^	Israel	Israeli Ministry of Health	8393	Gradient boosting with decision tree	0.90 auROC 95% CI: 0.892-0.905
Batista et al.^23^	Brazil	Brazilian Ministry of Health	235	Neural networks,RF,LR,SVM,Gradient boosting trees	AUC: 0.85; Sensitivity: 0.68; Specificity: 0.85; Brier Score: 0.16
Prefeitura et al.^24^	Brazil	Public Health Department of Florianópolis	3916	Random forest	Accuracy:0.66 (UI 95%0.62-0.69)Sensitivity:0.65 (UI 95%0.57-0.75);Specificity:0.66 (UI 95%0.60-0.70)
Mei et al.^14^	China	18 medical centers in 13 provinces in China	419	CNN, SVM, RF, MLP	AUC 0.92
Chen et al.^19^	China	Union Hospital, Wuhan, China	214	RF	Accuracy>95%
Li et al.^16^	USA	Public data	413	XGBoost	Sensitivity 92.5%;Specificity 97.9%
Avila et al.^25^	Brazil	Hospital Israelita Albert Einstein (HIAE - São Paulo, Brazil)	510	Naïve Bayes Classifier	Sensitivity and Specificity 76.7%

*Public data:https://github.com/yoshihiko1218/COVID19ML, KNHIS: Korean National Health Insurance Service, SVM: Support Vector Machines, LSVM: Linear support vector machine,NB: Naïve Bayes, RF: Random Forest, LR: Logistic Regression, KNN: K-nearest neighbors, ET: Extremely Randomized Trees, DT: Decision Tree, CNN: Convolutional Neural Networks, MLP: Multi-layer perceptron, LASSO: Least absolute shrinkage and selection operator, AUROC: Area under the receiver operating characteristic curve, AUC: Area under the curve

**Table 2:** Application of deep learning-based COVID-19 diagnosis

Authors	Data Sources	No. of Images	Type of Images	No. of Classes	Techniques	Type of model	Performances
Ardakani et al.^29^	Real-time data fromuniversity hospital	1020 (COVID19=510,non-COVID19=510)	CT	2 (COVID-19,non-COVID19)	AlexNet, VGG16, VGG-19,SqueezeNet, GoogleNet, MobileNet-V2, ResNet-18, ResNet-50, ResNet-101, Xception	Pre-trained model	Accuracy=99.51, Sensitivity=100, Specificity=99.02, Precision=99.27, AUC=99.4, NPV=100
Wu et al. 28	China Medical University, Beijing Youan Hospital	495 (COVID19=368, otherpneumonia=127)	CT	2 (COVID-19, other pneumonia)	ResNet50	Pre-trained model	Accuracy=76, Sensitivity=81.1, Specificity=61.5, AUC=81.9
Cifci 30	kaggle.com (benchmarkweb of dataset science)	5800	CT	2 (COVID-19, other pneumonia)	AlexNet, Inception-V4	Pre-trained model	Accuracy=94.74, Sensitivity=87.37, Specificity=87.45
Apostolopoulosand Bessiana^33^	COVID-19 X-ray imagedatabase^48^, Kaggledataset*,	1442 (COVID19=224,pneumonia=714,normal=504)	X-RAY	3 (COVID-19, pneumonia, normal)	VGG19, MobileNetv2, Inception, Xception, InceptionResNetv2	Pre-trained model	Accuracy=96.78, Sensitivity=98.66, Specificity=96.46
Loey et al. 34	COVID-19 X-ray imagedatabase^48^, Dataset*	307(COVID=69, normal=79,pneumonia_bac=79,pneumonia_vir=79)	X-RAY	4 (COVID, normal, pneumonia_ba, pneumonia_vir)	GAN, Alexnet, Googlenet, Resnet18	Pre-trained model	Accuracy=85.2, Precision= 80.6,
Hasan et al.^31^	COVID-19 Dataset*, SPIEAAPM-NCI Lung NoduleClassification Challenge Dataset	321 (COVID19=118,pneumonia=96,healthy=107)	CT	3 (COVID19, pneumonia, healthy)	QDE-DF	Customized Model	Accuracy=99.68
Amyar et al.^32^	COVID-CT^49^, COVID-19 CTsegmentation dataset*,a hospital named Henri Becquerel Center	1044 (COVID19=449, nonCOVID-19=595)	CT	2 (COVID19, nonCOVID-19)	EncoderDecoder withmulti-layerperceptron	Customized Model	Accuracy=86, Sensitivity=94, Specificity=79, AUC=93
Ozturk etal.^36^	COVID-19 X-ray image database 48, ChestX-ray8 50	1127 (COVID=127,no-finding=500,pneumonia=500)	X-RAY	3 (COVID, nofinding,pneumonia)	DarkNet	Customized Model	Accuracy=98.08, Sensitivity=95.13, Specificity=95.3, Precision=98.03, F1-Score=96.51
Rahimzadeh and Attar^35^	COVID-19 X-ray image database 48, RSNA Pneumonia Detection Challenge dataset 51	15085 (COVID19=180, pneumonia=6054, normal= 8851)	X-RAY	3 (COVID-19, pneumonia, normal)	ConcatenatedCNN	Customized Model	Accuracy=99.50, Sensitivity=80.53, Specificity=99.56

*Kaggle dataset: https://www.kaggle.com/andrewmvd/convid19-x-rays, Dataset: https://drive.google.com/uc?id=1coM7x3378fOu2l6Pg2wldaOI7Dntu1a, Covid-19 Dataset: https://radiopaedia.org, Archive, C.I. SPIE-AAPM-NCI Lung Nodule Classification Challenge Dataset: https://www.cancerimagingarchive.net, COVID-19 CT segmentation dataset: http://medicalsegmentation.com/covid19/ .Note: CT: computerized tomography; CNN: Convolutional Neural Network

**Table 3:** Challenges and perspectives of machine learning-based COVID-19 diagnosis

Challenges	Perspectives
Improve the accuracy of the AI diagnosis	Combine chest imaging with clinical symptoms, exposure history, and laboratory tests in the diagnosis of COVID-19
Reduce the false negative diagnosis rate	Provides spare capacity for CT and X-ray imaging scans with the advantages of rapid COVID-19 diagnosis.
